# Differentiation of Stem Cells from Human Exfoliated Deciduous Teeth into Retinal Photoreceptor-Like Cells and Their Sustainability *In Vivo*

**DOI:** 10.1155/2019/2562981

**Published:** 2019-02-14

**Authors:** Xiaoxia Li, Jing Xie, Yue Zhai, Tengjiaozi Fang, Nanquan Rao, Shuang Hu, Liping Yang, Yuming Zhao, Yixiang Wang, Lihong Ge

**Affiliations:** ^1^Department of Pediatric Dentistry, Peking University School and Hospital of Stomatology, National Engineering Laboratory for Digital and Material Technology of Stomatology, and Beijing Key Laboratory of Digital Stomatology, Beijing 100081, China; ^2^Department of Stomatology, Shenzhen Children's Hospital, Shenzhen 518026, China; ^3^Institute of Systems Biomedicine and Department of Ophthalmology, School of Basic Medical Sciences, Third Hospital, Peking University, Beijing 100191, China; ^4^Central Laboratory, Peking University School and Hospital of Stomatology, National Engineering Laboratory for Digital and Material Technology of Stomatology, and Beijing Key Laboratory of Digital Stomatology, Beijing 100081, China

## Abstract

Retinal degeneration is characterized by the progressive loss of photoreceptors, and stem cell therapy has become a promising strategy. Many studies have reported that mesenchymal stem cell transplantation can sustain retinal structure and prolong retinal functions based on two mechanisms. One is cell replacement, and the other is the paracrine action of stem cells. Cells from human exfoliated deciduous teeth (SHEDs) show characteristics typical of mesenchymal stem cells. They are derived from the neural crest and are a potential cellular source for neural regeneration in stem cell therapy. In this study, we explored the potential of SHEDs to be induced towards the retinal photoreceptor phenotype and to be sustainable in an animal model of retinal degeneration. A factor-cocktail protocol was used to induce SHEDs towards retinal photoreceptors for 24 days, and the characteristics of the induced cells were identified in terms of morphological changes, biomarker expression and subcellular distribution, and calcium influx. SHEDs were labeled with firefly luciferase for *in vivo* tracking by bioluminescent imaging and then transplanted into the subretinal space of mice. Our results showed that SHEDs successfully transdifferentiated into photoreceptor-like cells, which displayed neuron-like morphology, and expressed specific genes and proteins associated with retinal precursors, photoreceptor precursors, and mature photoreceptors. In addition, calcium influx was significantly greater in the retinal-induced than in noninduced SHEDs. *In vivo* tracking confirmed at least 2 weeks of good survival by bioluminescent imaging and 3 months of sustainability of SHEDs by histological analysis. We conclude that SHEDs have the potential to transdifferentiate into retinal photoreceptor-like cells *in vitro* and maintain good viability *in vivo* after transplantation into mice with a normal immune system. This demonstrates preliminary success in generating photoreceptor-like cells from SHEDs and applying SHEDs in treating retinal degeneration.

## 1. Introduction

Retinal degeneration associated with photoreceptor loss causes visual impairment and even untreatable blindness, affecting millions of people. Human retinal neurons have a limited ability to repair themselves or regenerate, especially the photoreceptors (rods and cones) which are terminal sensory neurons connected to the first cranial nerve (optic nerve). Today, stem cell therapy is a prospective strategy for treating retinal degeneration [1, 2], and finding an ideal source of stem cells for transplantation is a key issue for this field.

Different approaches to retinal regeneration have been explored. One important strategy is to use cells or tissues derived *in vitro* to replace injured retinal cells by transplantation. Photoreceptors derived from human embryonic stem cells (ESCs) or induced pluripotent stem cells (iPS cells) and engineered retinal tissues have shown great potential to repair the structure and function of damaged retinal tissues in animal models of retinal degeneration. However, the ethical controversy and the immunological rejection associated with ESCs or the risk of genetic mutations associated with iPS cells prevents their clinical application. Therefore, human adult stem cells without these concerns are emerging as a promising approach.

Mesenchymal stem cells (MSCs) are adult stem cells that can be isolated from many tissues, such as bone marrow, as well as deciduous teeth. They possess multilineage differentiation potential including neural fate and have paracrine trophic and immunomodulatory effects [3, 4]. Stem cells from human exfoliated deciduous teeth (SHEDs) possess characteristics typical of MSCs including neural differentiation [5]; they express ESC markers [6] and have immunomodulatory action [7]. Reports have confirmed that transplantation of human bone marrow MSCs prolong retinal function in animals with retinal degeneration [8–10]. The reparative activity of MSCs in restoring retinal function includes two mechanisms: one is cell replacement, based on neural differentiation, and the other is their paracrine actions that have favorable effects such as neurotropic protection, immunomodulation, antiapoptosis, anti-inflammation, and regulation of angiogenesis [11]. Theoretically, since they originate from the neural crest, SHEDs are likely to have a better capacity for neural differentiation than are other kinds of MSCs. It has been confirmed that SHEDs secrete neurotrophic factors, cytokines, and chemokines which favor neural repair [12–14], as well as anti-inflammatory activity [15] and regulation of angiogenesis [16], but whether SHEDs can differentiate into retinal neurons is unknown. In addition, there are no reports on using SHEDs in treating retinal disease as stem cell therapy. So, in this study, we first aimed to investigate the potential of SHEDs to differentiate into retinal photoreceptors and then further explored their sustainability and viability *in vivo* as a preliminary step toward preclinical trials.

## 2. Materials and Methods

### 2.1. SHED Culture and Identification

SHEDs were a gift from the Oral Stem Cell Bank of Beijing, Tason Biotech Co. Ltd. The culture medium was alpha-modified Eagle's minimum essential medium (*α*-MEM, Gibco BRL, Grand Island, NY, USA) supplemented with 10% fetal bovine serum (Gibco BRL), 1% penicillin/streptomycin, and 2 mM glutamine under 100% humidity and 5% CO_2_ at 37°C. Flow cytometry was performed to assess the quantity of surface markers of SHEDs with antibodies specific for CD90, CD105 (BioLegend, San Diego, CA, USA), CD73, CD146, CD34, and CD45 (BD Biosciences, San Jose, CA, USA). Immunofluorescence was also used to identify the neural markers *β*-III tubulin and nestin and the glial marker GFAP in SHEDs. Besides, we quantified the positive rates of *β*-III tubulin, nestin, and GFAP in SHEDs using flow cytometry. Details of the flow cytometry and immunofluorescence are in the following paragraphs.

### 2.2. Retinal Differentiation and Morphological Observation

SHEDs were induced according to a previously established protocol with modification [17–19]. In brief, SHEDs at passages 3-5 were recruited for two-step induction. In step one, floating culture (2 × 10^5^ cells/mL) in a low-attachment dish (Nest, Wuxi, China) for 3 days was carried out to obtain neuro-like spheres in Dulbecco's modified Eagle's medium (DMEM)/F12 supplemented with 1 × B27 (Invitrogen), 10% knockout serum replacement (Gibco BRL), 1 ng/mL noggin (PeproTech, Rocky Hill, NJ, USA), 1 ng/mL Dickkopf-related protein 1 (DKK-1; PeproTech), and 5 ng/mL insulin-like growth factor 1 (IGF-1; PeproTech).

In step 2, neurospheres were collected and dissociated after 3 min of Accutase incubation (STEMCELL Technologies, Vancouver, BC, Canada) at 37°C. The cell suspension was then transferred to dishes or coverslips coated with Matrigel (Corning, NY, USA) at 2 × 10^4^ cells/cm^2^. During the previous 7 days (days 3 to 10), cells were treated with 1 × B27 supplement, 1 × N2 supplement (Invitrogen, Carlsbad, CA, USA), 100 ng/mL noggin (PeproTech), 10 ng/mL DKK-1 (PeproTech), 50 ng/mL IGF-1 (PeproTech), and 20 ng/mL basic fibroblast growth factor (bFGF, PeproTech) in DMEM/F12. During the final 14 days of induction, the culture medium was switched to 1 × N2 supplement, 1 × insulin-transferrin-selenium supplement (Gibco BRL), 10 ng/mL noggin, 10 ng/mL DKK-1, 10 ng/mL IGF-1, 20 ng/mL bFGF, 20 ng/mL sonic hedgehog (Shh; PeproTech), 40 ng/mL 3,3′,5-triiodo-L-thyronine (T3; Sigma), and 500 nM all-trans retinoic acid (Sigma-Aldrich) in DMEM/F12. The medium was changed every 3 days. Changes of morphology were observed under an inverted phase-contrast microscope (Olympus, Tokyo, Japan).

### 2.3. Flow Cytometry Analysis

Cells were collected and washed twice in buffer solution (PBS containing 2% FBS). For the cell surface markers, the cells were incubated with fluorescein-isothiocyanate- (FITC-) conjugated or phycoerythrin- (PE-) conjugated primary antibodies at 4°C and protected from light. For the intracellular biomarkers, cells were fixed in 4% paraformaldehyde (PFA) for 20 min and washed with the buffer solution, then pretreated with 0.5% (*v*/*v*) Tween 20 (Sigma-Aldrich, St. Louis, MO, USA) for 15 min and washed with the buffer solution again. For indirect staining, the cells were incubated with unconjugated primary antibodies at 4°C followed by incubation with FITC- or PE-conjugated secondary antibodies at 4°C with protection from light. Finally, samples were washed and kept in the dark prior to analysis. Single staining flow cytometry was performed using EPICS XL (Beckman Coulter, Kraemer Boulevard Brea, CA, USA) with EXPO032 ADC XL 4 Color software (Beckman Coulter). Double staining flow cytometry was performed using BD FACSCalibur (BD) with CellQuest software (BD). The primary antibodies used are detailed in the Supplementary Material (Table S1).

### 2.4. Immunofluorescence

Coverslips were rinsed with 0.01 M phosphate-buffered saline (PBS) and fixed in 4% PFA in PBS for 20-30 min at room temperature. The fixed cultures were incubated in 1% BSA containing 0.1% Triton X-100 for 1 h and then incubated with primary antibodies (Supplementary Table S1) at 4°C overnight. The cells were incubated with the secondary antibodies Alexa Fluor 488/594-conjugated AffiniPure goat anti-rabbit IgG (H + L) (Proteintech, Wuhan, China) and Alexa Fluor 488/594-conjugated AffiniPure goat anti-mouse IgG (H + L) (Proteintech) for 1 h at room temperature in the dark and finally counterstained with 4′,6-diamidino-2-phenylindole (DAPI). The cells were viewed under a fluorescence microscope (Olympus) or a laser-scanning confocal microscope (Zeiss, Jena, Germany). In each immunofluorescence experiment, we set up negative controls in which the primary antibody was not used or noninduced SHEDs were used for staining of retinal differentiation biomarkers to ensure that the procedures were correct exclude the possibility of false-positive results. Antibodies were purchased from reliable manufacturer such as Abcam, CST, Millipore, BioLegend, and Proteintech, and we applied them in experiments according to the manufacturers' instructions and with reference to published manuscripts in which the same antibodies have been used. Furthermore, we stained frozen sections of retinas from wild-type mice with key retina-specific antibodies such as rhodopsin, opsin, recoverin, and PKC-*α* as positive references, and they were in the right locations in the retina (Figure S1).

### 2.5. Real-Time Reverse-Transcription Polymerase Chain Reaction Analysis

Total mRNA was extracted using TRIzol reagent (Invitrogen) according to the manufacturer's instructions. Total RNA was converted to cDNA using a reverse transcriptase kit (Promega, Madison, WI, USA). Glyceraldehyde-3-phosphate dehydrogenase (GAPDH) was used as an internal control. The primer sequences are listed in Supplementary Table S2. Quantitative polymerase chain reaction (qPCR) was carried out in triplicate in 96-well plates using a 7900HT Fast Real-Time system (Applied Biosystems, Foster City, CA, USA). The comparable cycle threshold method (2^-⊿⊿CT^) was used to calculate the relative expression levels of the target genes.

### 2.6. Calcium Imaging Assay

Intracellular Ca^2+^ transients were monitored using the Ca^2+^ indicator fluo-4-acetoxymethyl ester (Fluo-4 AM; Invitrogen). Both control and induced cells (day 24) were incubated for 30 min at 37°C in Hank's balanced salt solution (HBSS; Invitrogen) containing 5 *μ*M Fluo-4 AM and 0.05% F-127 (Sigma-Aldrich). Cells were washed three times with HBSS and then incubated in extracellular fluid (in mM: 150 NaCl, 5 KCl, 2.5 CaCl_2_, 1 MgCl_2_, 10 Hepes, and 10 D-glucose, pH 7.4).

Fluorescence images were immediately captured on a Leica laser scanning confocal microscope (TCS SP8, Wetzlar, Germany) at an excitation wavelength of 488 nm and emission at 510 nm under a 40x objective lens. Images were captured every 5 s for 5 min with 60-100 cells in each microscopic field, and a final concentration of 10 *μ*M glutamate (Sigma-Aldrich), or a high concentration of KCl (100-150 *μ*M, Sigma-Aldrich) was gently perfused onto the cells at 25 s using an Eppendorf micropipettor. At least 3 samples of both SHEDs on day 0 and induced SHEDs on day 24 were assessed under only one condition. The primary data were analyzed, and pseudo-color images were processed by selecting an appropriate color bar with Leica Application Suite X software. The change of cellular fluorescence intensity represented the drug-induced intracellular Ca^2+^ transient and was calculated for each cell using the formula: % fluorescence change = (*F*–*F*_baseline_)/*F*_baseline_ × 100%.

### 2.7. SHED Labeling with Firefly Luciferase and CM-Dil

To trace transplanted cells in *vivo*, SHEDs were labeled with firefly luciferase which enabled live cells to emit a detectable bioluminescent signal. Lentiviral vector particles Ubi-MSC-SV40-Luc-IRES (Gene, Shanghai, China) were transfected into SHED genes at a multiplicity of infection of 10. In order to select SHEDs stably expressing luciferase, 0.25 *μ*g/mL puromycin (Sigma-Aldrich) was added to the medium for 3 passages to kill SHEDs that were not labeled with luciferase. *In vitro* bioluminescence signals were collected by a 96-well microplate luminescence detector Centro XS3 LB 960 (Berthold Technologies, Bad Wildbad, Baden-Württemberg, Germany) after incubation with lysis buffer (Bright-Glo™ Luciferase Assay System, Promega) for 5 min. Luciferase intensity was normalized to the OD value of the Cell Counting Kit-8 assay (Dojindo Laboratories, Kumamoto, Japan) which indicated the number and viability of cells. Differentiated SHEDs on days 14-17 were used for transplantation. All luciferase-SHEDs were labeled with the cell membrane dye chloromethyl-benzamidodialkylcarbocyanine (CM-Dil; Invitrogen) 10-12 h before transplantation according to the manufacturer's instruction in order to identify them in histological examination.

### 2.8. Animals and Subretinal Transplantation of SHEDs

We used the well-established model of slow retinal degeneration, *RPGR*-knockout C57/BL6J mice, which were a kind gift from Prof. Yang Liping (Third Hospital of Peking University). These mice undergo retinal degeneration very slowly from the age of 2 months, with a substantial decrease of photoreceptors and their biomarkers rhodopsin and opsin by 6 months and a significant reduction of outer nuclear layer thickness and loss of rhodopsin and opsin expression at 12 months [20, 21]. This study was approved by the Laboratory Animal Welfare Ethics Branch of the Biomedical Ethics Committee of Peking University (LA2018240). All surgical interventions and animal care were in accord with the Guide for the Care and Use of Animals of Peking University Health Science Center. Animals were housed in a regulated environment (22 ± 2°C, 55 ± 5% humidity, and a 12 h light : 12 h dark cycle) with *ad libitum* food and water.

We chose 3-4 months as the transplantation age, before the peak of degeneration. CM-Dil-stained luciferase-SHEDs were transplanted into the experimental group (*n* = 6), or the balanced medium was injected into the control group (*n* = 6) under an ophthalmic operating microscope (Topcon, Beijing, China). The pupils were dilated with 1% tropicamide 30 min before surgery. Ketamine (100 mg/kg) and xylazine (10 mg/kg) were injected i.p. for general anesthesia, and dicaine hydrochloride eye drops were used for local anesthesia. Intraocular pressure was first reduced by a puncture at the edge of the cornea. A 33 G microinjector needle on a 5 *μ*L syringe (Hamilton, Bonaduz, Switzerland) was inserted into the temporal side of the eye through the cornea, conjunctiva, and sclera, finally reaching the subretinal space, and forming a self-sealing wound. About 1 *μ*L cell suspension (2 × 10^4^ cells/*μ*L) or 1 *μ*L of balanced medium containing sodium fluorescein but not serum was injected into the subretinal space. Successful transplantation was confirmed by the presence of yellow-green fluorescent liquid in the fundus. Ofloxacin eye drops were applied topically to the eye 3 times/day for 3 days after injection.

### 2.9. *In Vivo* Bioluminescence Imaging

Bioluminescence images of mice at 7, 14, 21, and 28 days after surgery were acquired with an IVIS Lumina III Series instrument (Caliper Life Sciences, MA, USA). Mice received 15 mg/mL D-luciferin (150 mg/kg, i.p.; Promega) 5 min before general anesthesia with 4% chloral hydrate (400 mg/kg). Bioluminescence images were collected every minute for a total of 15-20 min and analyzed with Living Image 4.5.3 software (PerkinElmer, Shanghai, China). The luminescence intensity in regions of interest from each image was quantified to assess the viability of implanted cells. In addition, 200 *μ*L of 1 × 10^4^ luciferase-SHED suspension or *α*-MEM in an Eppendorf tube containing 300 *μ*g/mL D-luciferin was imaged with the IVIS Lumina III Series instrument as a positive or a negative control.

### 2.10. Histological Analysis

Eyes were removed and immersed in 4% PFA for 4 h. Then, they were infiltrated with 20% sucrose for at least 24 h and 30% for ~12 h until they did not float, after which they were embedded in O.C.T. Compound (Sakura, Torrance, CA, USA) with the vertical meridian of the eye through both the optic nerve and the injection site and in the cutting orientation. Sections were cut at 5 *μ*m and stained with DAPI or hematoxylin and eosin.

### 2.11. Statistical Analysis

All experiments included at least 3 biological replicates and experimental replicates. qRT-PCR, Ca^2+^ influx peaks, relative luciferase intensity *in vitro*, and bioluminescence intensity *in vivo* were compared using two-tailed *t*-tests for independent samples, and multiple comparisons were performed using one-way ANOVA in GraphPad Prism 7 (La Jolla, CA, USA). Statistical significance was set at *p* < 0.05. Data are shown as mean ± SD.

## 3. Results

### 3.1. Characteristics of SHEDs

**Figure 1:**
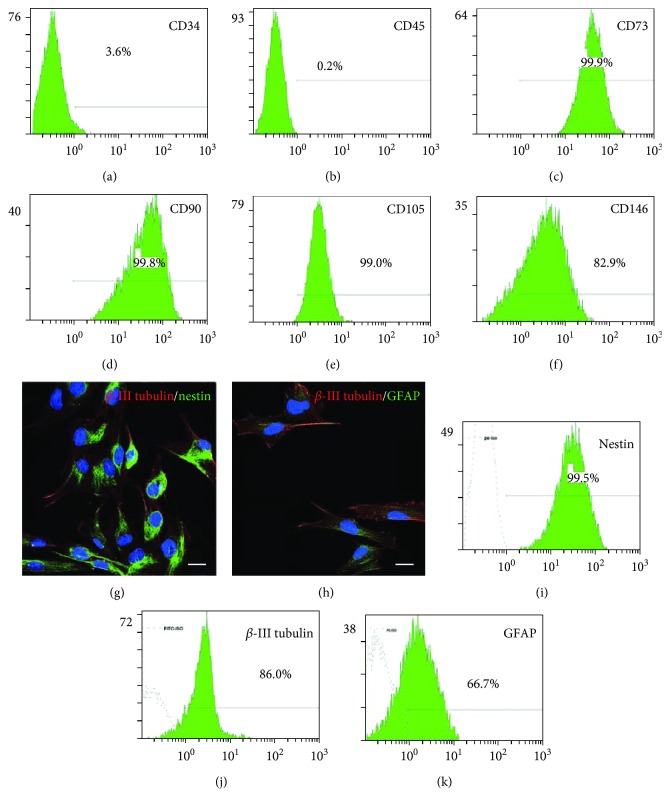
Identification of SHEDs. (a)–(f) Flow cytometry plots showing that the cultured SHEDs were positive for the mesenchymal markers CD73, CD90, CD105, and CD146, but negative for the hematopoietic marker CD34 and the common leukocyte antigen CD45. (g)-(h) Representative photomicrographs of SHEDs expressing the neural stem cell markers nestin and *β*-III tubulin, and the glia-specific marker GFAP. *Scale bars: 20 μm.* (i)–(k) Flow cytometry plots showing the percentages positive for nestin, *β*-III tubulin, and GFAP.

SHEDs were spindle-shaped, resembling fibroblasts, adhered to a plastic surface, and proliferated rapidly. Flow cytometry showed that cultured SHEDs expressed the typical mesenchymal markers CD73 (99.9%), CD90 (99.8%), CD105 (99.0%), and CD146 (82.9%) and were negative for the hematopoietic marker CD34 and the common leukocyte antigen CD45 (Figures 1(a)–1(f)). Immunostaining and flow cytometry demonstrated that SEHDs expressed the neural markers nestin (99.5%) and *β*-III tubulin (86%) and the glial marker GFAP (66.7%) (Figures 1(g)–1(k)).

### 3.2. Retinal Differentiation and Photoreceptor-Like Morphology of SHEDs

**Figure 2:**
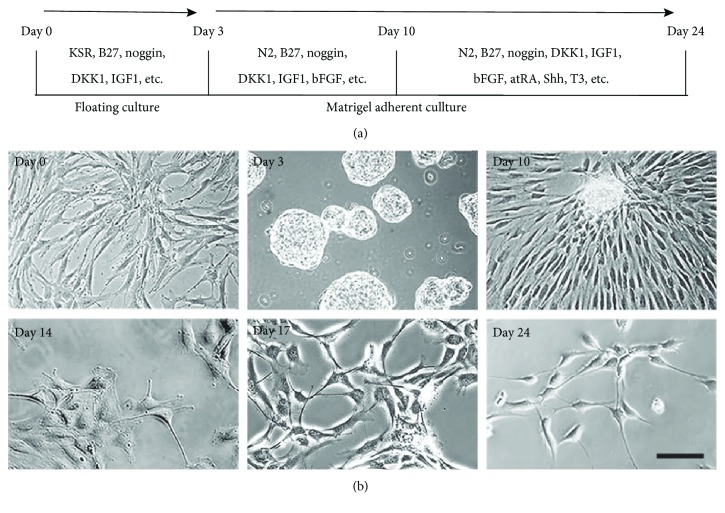
Morphological changes during *in vitro* retinal differentiation. (a) Retinal differentiation protocol. (b) Morphological changes of SHEDs during retinal induction. On day 3, large floating neurosphere-like masses were observed. After retinal cell (factor cocktail) induction on Matrigel, the cells became larger and extended cytoplasmic processes like neurites, cross-linking with adjacent cells about 14 days post-induction. At the end of induction (day 24), cells displayed the morphology of neuron-like cells. *Scale bar: 50 μm.*

The induction protocol is shown in Figure 2(a). Inhibition of BMP and WNT signaling promotes anterior neural plate development and retinal fate differentiation [17, 22, 23]. DKK-1 antagonizes Wnt/*β* catenin signaling, and noggin inhibits BMP signaling; these two key factors are essential for inducing SHEDs toward retinal photosensory cells. B27 and N2, which play essential roles in retinal cell differentiation [24], were present throughout the differentiation process. Other factors have specific functions in retinal differentiation; for example, Shh promotes telencephalic differentiation and the generation of retinal precursors [25], and T3 and retinoic acid promote the differentiation of photoreceptors in human fetal retinal cultures [26]. We tested different factor cocktails and optimized them to finally establish a relatively efficient and stable induction protocol.

After 3 days in floating culture, SHEDs formed neurosphere-like clusters. After prolonged induction in step 2, SHEDs migrated out of the sphere and displayed a neural cell morphology with enlarged, rounded cell bodies and short or long processes cross-linking with neighboring cells (Figure 2(b)).

### 3.3. Expression Profiles of Genes and Proteins in Induced SHEDs

**Figure 3:**
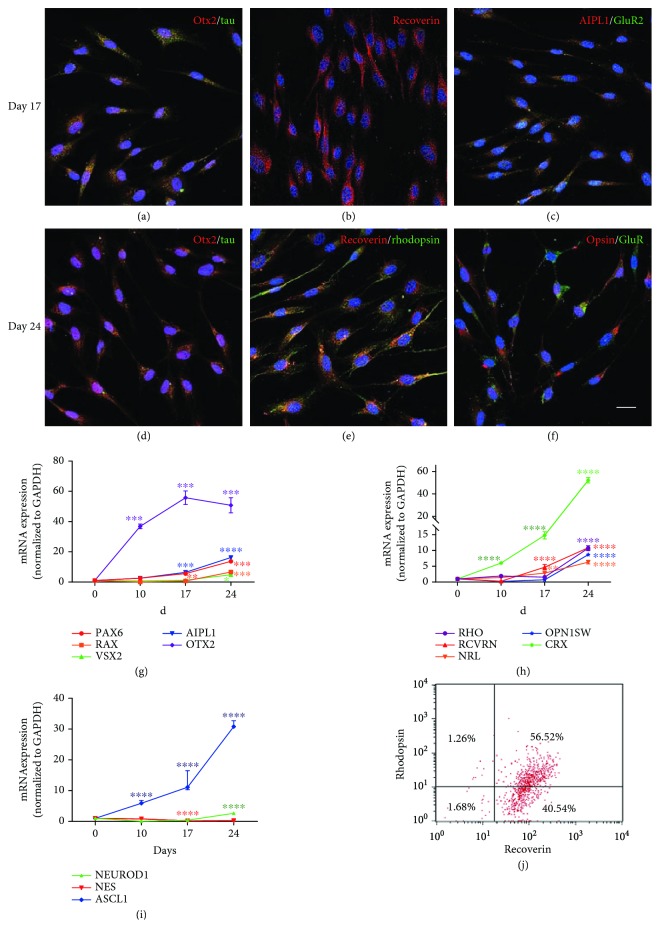
Characteristics of expression profiles of gene and protein biomarkers in photoreceptor-like cells derived from SHEDs after retinal induction. (a)–(c) Immunofluorescence images showing SHEDs expressing the retina-related neural markers tau (a) and GluR2 (c), the retina-related neural markers Otx2 (a) and AIPL1 (c), and the photoreceptor precursor marker recoverin (b) on day 17. (e)-(f) The rod marker rhodopsin (e) and the cone marker opsin (f) were detected on day 24, as were Otx2 (d), tau (d), GluR (f), and recoverin (e). *Scale bars: 20 μm.* (g)–(i) q-PCR analysis showing that relative gene expression of the retinal progenitor markers PAX6, RAX, and VSX2 was upregulated, along with the retinal neural markers OTX2 and AIPL1 (g). The photoreceptor precursor markers RCVRN, CRX, and NRL showed an increasing trend, and the rod marker RHO and the cone marker OPN1SW were highly expressed at the final stage of induction (h). In addition, there was an increasing trend in the expression of the proneural markers NEUROD1 and ASCL1, but a decreasing trend in the expression of the neural crest cell marker NES (i) (^∗^ *p* < 0.05, ^∗∗^ *p* < 0.01, ^∗∗∗^ *p* < 0.001, and ^∗∗∗∗^ *p* < 0.0001). (j) Flow cytometry analysis showing the percentages positive for recoverin, rhodopsin, or both of them.

We carried out qPCR and immunofluorescence to assess the changes in expression of biomarkers in induced SHEDs during differentiation. After 17 days of induction, SHEDs were positive for the pan-neural markers tau and GluR2, the retinal-related neural markers Otx2 and AIPL1, and the photoreceptor precursor marker recoverin (Figures 3(a)–3(c)). On the final stage of induction (day 24), SHEDs still expressed recoverin, Otx2, tau and GluR2 (Figures 3(d)–3(f)) and were positive for the rod marker rhodopsin (Figure 3(e)) and the cone marker opsin (Figure 3(f)). Noninduced SHEDs did not express these biomarkers (Figure S1). Immunonegativity for GFAP in induced SHEDs excluded the possibility of glial differentiation (Figure S3).

The expression of genes was consistent with the expression of proteins. qPCR showed that the early retina-related transcription factor PAX6 was upregulated after day 10 with fold changes of ~14, while the expression of RAX remained at a low level until day 24 (Figure 3(g)). VSX2, a specific marker for retinal neural epithelium differentiation but not retinal pigment epithelium, was upregulated until day 24 (Figure 3(g)). Another two retina-related neural markers, OTX2 and AIPL1, increased from day 10, and OTX2 reached a peak on day 17 with a fold change of ~60 (Figure 3(g)). The expression levels of the markers of photoreceptor precursor specification recoverin increased early on day 10, and CRX was highly expressed after day 10 with fold changes of ~50; NRL was not expressed until day 24 (Figure 3(h)). The expression of OPN1SW for cones and RHO for rods reached an abrupt peak with fold changes of ~10 at 24 days (Figure 3(h)). Besides, we found that the neural crest gene marker NES showed a downward trend in expression (Figure 3(i)). Genes associated with pan-neural differentiation (ASCL1 and NEURODD1) showed an upward trend in expression, that of ASCL1 being increased ~30 times on day 24 (Figure 3(i)). Quantitative analysis showed that 97.1% of the induced SHEDs on day 24 expressed recoverin, 57.8% expressed rhodopsin, and 56.52% coexpressed recoverin and rhodopsin by flow cytometry (Figure 3(j)).

The pluripotent marker SSEA4 was expressed by ~57.9% of noninduced SHEDs, but they did not express it after day 14 (a–c, Figure S3). Also, there was a decreasing tendency in the expression of the pluripotent genes NANOG and MYC, but an increasing tendency in SOX2 and POU5F1 (d, Figure S3). It has been reported that upregulation of SOX2 and POU5F1 together disrupts the self-renewal of ESCs and promotes their differentiation, including into neuroectoderm [27, 28], which also occurs after knockdown or downregulation of NANOG and MYC [29, 30]; this is consistent with our results. Besides, differentiated SHEDs proliferated at a very low rate: the cell density stopped increasing after day 17. These results also suggest that retina-induced SHEDs are not like the rapidly proliferating multipotent stem cells.

### 3.4. Photoreceptor-Like Cells Derived from SHEDs after Retinal Induction Responded to the Neurotransmitter Glutamate and High K^+^

**Figure 4:**
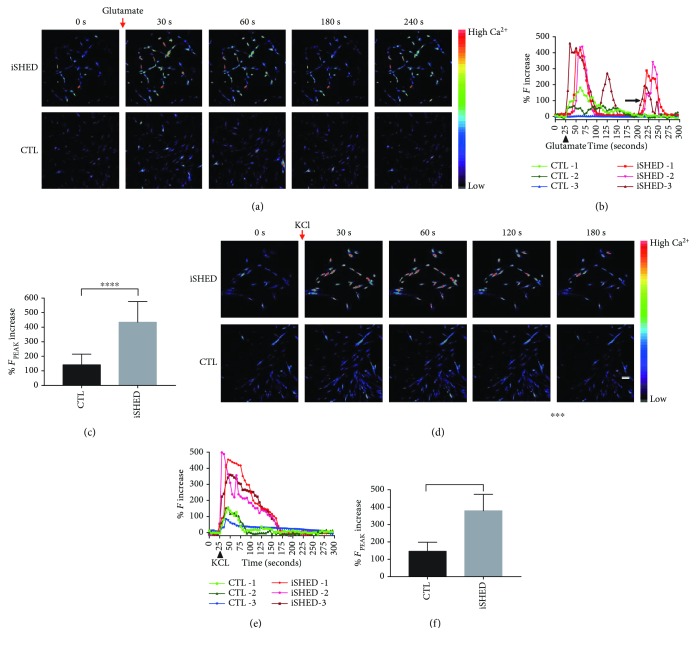
Ca^2+^ activity of SHEDs on day 0 (control, CTL) and day 24 (iSHEDs). (a, d) Representative pseudo-color images showing a greater fluorescence increase in induced cells than in controls after exposure to glutamate (a) and high K^+^ (d) (color code denotes Ca^2+^ level). (b, e). Representative Ca^2+^ transient profiles with time displaying higher Ca^2+^ influx peaks in the induced cells than in the controls ((b) for glutamate, (e) for KCl); the arrow indicates second or third Ca^2+^ influx peaks in induced SHEDs (b). (c, f) Histograms showing the fluorescence intensity at the peak time was higher in the induced cells than in controls ((c) for glutamate, (f) for KCl) (^∗∗∗^ *p* < 0.001 and ^∗∗∗∗^ *p* < 0.0001).

Ca^2+^ activity plays critical roles in neural function, including the regulation of neurite elongation [31], synaptic transmission and plasticity [32, 33], and cell survival [34]. Glutamate is a neurotransmitter which generally excites neurons. High K^+^ depolarizes the cell membrane and activates voltage-gated Ca^2+^ channels. To assess Ca^2+^ activity upon stimulation with the neurotransmitter glutamate or high K^+^, we conducted Ca^2+^ imaging of induced SHEDs on day 24 and noninduced control cells on day 0. The time series of pseudo-color fluorescence images (until 240 s for glutamate stimulation when the second Ca^2+^ influx peaks emerged, and until 180 s for KCl stimulation, during which the unique Ca^2+^ influx peaks appeared) showed that both noninduced and induced SHEDs responded to glutamate and KCl exposure with brighter fluorescence after stimulation, but the induced cells displayed a greater fluorescence increase than the controls did (Figures 4(a) and 4(d)). The response kinetics of fluorescence intensity against time varied among cells, and the induced SHEDs displayed a stronger response upon both glutamate and KCl stimulation (Figures 4(b) and 4(e)). The fluorescence intensity at the peak time in the induced cells was significantly greater than in control cells upon both glutamate and high K^+^ stimulation (Figures 4(c) and 4(f)).

We defined the cells whose fluorescence peaked after drug exposure as positive cells. In the glutamate experiment, the majority of induced cells (>81%) showed a rapid and robust increase in fluorescence intensity after addition of 10 *μ*M glutamate, reaching a peak at 30-80 s and then decaying slowly. However, only 18-20% of control cells showed a positive response, and the rare peaks were very low. On average, induced SHEDs showed Ca^2+^ influx peaks 3 times higher than control SHEDs did. Some induced cells upon glutamate exposure displayed second or even third peaks (Figure 4(b)). In the KCl experiment, ~78% of induced cells and ~11.6% control cells showed a positive response. On average, the peak Ca^2+^ influx in induced SHEDs was 70 s longer and ~2.6 times higher than that in control SHEDs.

### 3.5. Sustainability and Survival of SHEDs *In Vivo*

**Figure 5:**
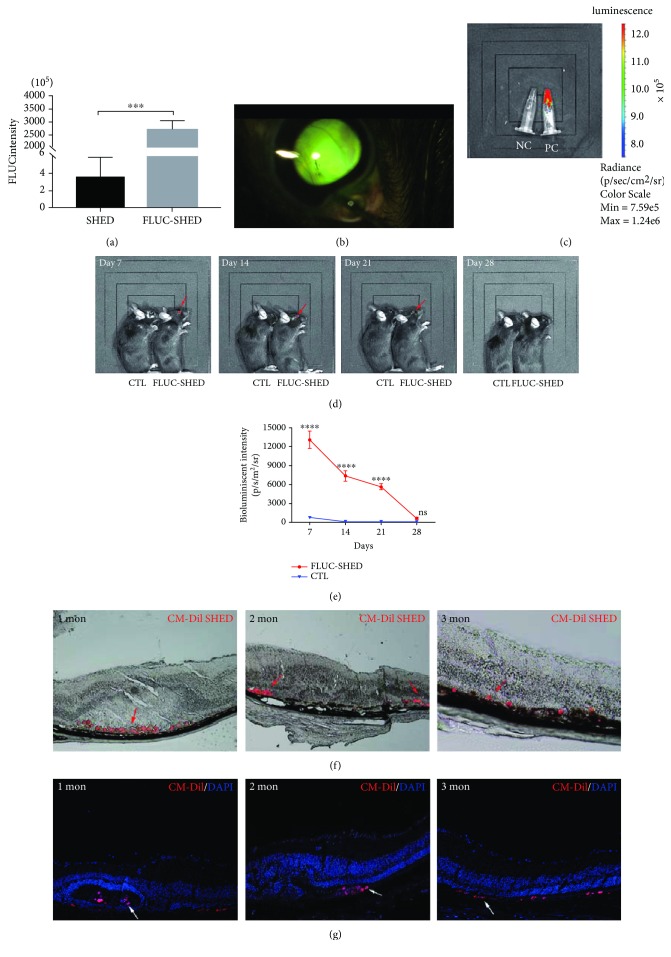
Sustainability and survival of SHEDs *in vivo*. (a) Relative fluorescence intensity was significantly higher in luciferase-SHEDs than in untransfected SHEDs. (b) Photograph of the subretinal space with transplanted luciferase-SHEDs. (c) Bioluminescence imaging of Eppendorf tubes containing luciferase-SHEDs (positive control, PC) emitting a strong bioluminescent signal while negative control SHEDs (NC) displayed no signal. (d) *In vivo* bioluminescent imaging of mice at 7, 14, 21, and 28 days after transplantation. A signal was emitted from the eye area specifically on day 7, 14, and 21 (red arrows). (e) Line chart showing the bioluminescent signal of the luciferase-SHEDs group declined rapidly with time, while the control group signal remained low (^∗^ *p* < 0.05; ^∗∗∗∗^ *p* < 0.0001). (f)-(g). Brightfield images (f) and fluorescent images (g) displaying SHEDs stained red with CM-Dil (red arrows for (f) and white arrows for (g)) in the subretinal space 1, 2, and 3 months after transplantation. *Scale bar: 50 μm*.

The bioluminescent signal in luciferase-SHEDs *in vitro* was prominently higher than in unlabeled SHEDs (Figure 5(a)), confirming stable labeling with firefly luciferase. Luciferase-SHEDs were successfully transplanted into the subretinal space as shown by the yellow-green fluorescence (Figure 5(b)). Previous studies have confirmed that cells stably expressing luciferase show a linear, positive relationship with cell numbers, and integration of the exogenous luciferase gene does not change cell properties [35–37]. In this study, a positive control with a strong bioluminescent signal and a negative control without any such signal were included (Figure 5(c)). Differentiated SHEDs on days 14-17 were used for transplantation when photoreceptor precursors appeared, and the cells had very good viability. Terminally differentiated cells are not usually suitable for transplantation. To observe the survival of SHEDs in the subretinal space, bioluminescence imaging was performed weekly after transplantation (Figures 5(d) and 5(e)). In the luciferase-SHED group, the bioluminescent signal specifically emitted from the eye area was most robust on day 7, and then declined gradually until day 21, and finally disappeared on day 28. In the control group, mice displayed no bioluminescent signals at any time point, as expected. Histological analysis showed that transplanted CM-Dil-labeled SHEDs were present in the subretinal space, with round cell bodies, at 1, 2, and 3 months after transplantation (Figures 5(f) and 5(g)). No tumors were revealed by histological analysis after transplantation of differentiated SHEDs (Figure S4). Considering about the low proliferation rate and the decreased expression of pluripotent markers in induced SHEDs *in vitro*, it is quite safe to use induced SHEDs for transplantation in mice.

## 4. Discussion

Here, we demonstrated that SHEDs have the potential to differentiate into photoreceptor-like cells. They formed neurosphere-like masses, which are characteristics of neural stem cells. When cultured on coated soft surfaces, they displayed neuronal morphology. In addition, the expression profiles of genes and proteins further confirmed the retinal photosensory properties of induced SHEDs at the molecular level. Ca^2+^ activity revealed that induced SHEDs possessed more functional glutamate receptors on their membrane and more activated voltage-gated Ca^2+^ channels to mediate neurotransmission, which are important traits of neurons.

During the process of induction, genes and proteins associated with retina-related transcription and photoreceptor specification were upregulated, while genes and proteins associated with pluripotent, mesenchymal features were downregulated. The expression profiles with time were similar to those of ESCs and iPS cells during differentiation towards photoreceptors [18]; during induction, SHEDs gradually lost their mesenchymal and came to resemble retinal photoreceptor cells. It is known that manipulation of signaling factors in the culture medium can control the time course of retinal differentiation in human ESCs [18], and different studies have reported different periods of CRX expression ranging from 1 to 13 weeks [17, 19, 24]. We also found differences in the time at which the same category of markers were expressed. For example, PAX6 was expressed on day 10 but RAX was expressed on day 24 in PCR, even after the photoreceptor precursor markers RCVRN and CRX appeared. This may be due to the influence of subtle variations of the signaling factors in the culture medium, or differences in the sensitivity of different genes to signaling factors. Furthermore, although the induction procedure largely mimicked the natural differentiation process during embryonic development, this was artificial induction of adult stem cells *in vitro*, and there may be a difference between artificial induction and natural embryonic development.

The induced cells showed greater Ca^2+^ influx, higher intracellular Ca^2+^ peaks, and higher number of positive cells in response to glutamate and KCl stimulation than noninduced control cells did. Our results were similar to those of previous studies. Neural cells transdifferentiated from bone marrow-derived MSCs show increasing cytoplasmic Ca^2+^ levels induced by KCl or glutamate [38, 39]. When induced toward a neural fate, bone marrow-derived MSCs gain membrane properties characteristic of neurons, such as voltage-gated Na^+^ channels, voltage-gated Ca^2+^ channels, and functional glutamate receptors [40]. Our results suggest that the photoreceptor-like cells derived from SHEDs are functional neurons that possess glutamate receptors and active voltage-gated Ca^2+^ channels and maintain good structural integrity of the cell membrane.

iPS cell-derived photoreceptor precursors express functional ionotropic glutamate receptors and respond to glutamate stimulation with Ca^2+^ influx [41], and rod precursors derived from newborn retina or iPS cells show Ca^2+^ responses to glutamate or KCl stimulation [42]. Bipolar, horizontal, and ganglion cells also express glutamate receptors [43–45]. We found that a small population of induced SHED cells expressed the bipolar marker PKC-*α* on days 14-17 but disappeared by day 24 (Figure S2). Therefore, the Ca^2+^ imaging results alone do not exclude the possibility that other kinds of retinal neural cells as well as rod-like and cone-like cells were generated. Further studies are needed to comprehensively identify all the photoreceptor-like cells that can be induced.

The expression of gene or protein biomarkers in cell cultures is an important indicator for cell-type classification, but is not sufficiently informative to define a specific cell type. The comprehensive morphological, metabolic, and functional characteristics of cells must be taken into account. For example, the expression of biomarkers in cultured adipocytes reflects the expression patterns of adipose tissue in developing mice between birth and weaning, but not adult mice [46]. In this study, we confirmed the expression of photoreceptor-related gene and protein biomarkers in a pattern similar to embryonic development, and this in a part reflects the functional changes of SHEDs after retinal induction. Next, we used calcium imaging, which reflects the physiological properties of neural cells, and found an increase in calcium influx in retina-induced SHEDs. Thus, it is reasonable to conclude that photoreceptor-like cells were generated in our protocol.

MSCs are heterogeneous, containing subpopulations differing in morphology (spindle-shaped, large flat, and small round cells), cellular markers, proliferation rates, and potential for multilineage differentiation [47]. Here, we found heterogeneity among SHEDs both before and after induction. For example, not all SHEDs (~66.7%) expressed GFAP on day 0, and ~57.8% of induced SHEDs expressed the photoreceptor marker rhodopsin on day 24 as indicated by flow cytometry. Heterogeneity was also found in calcium imaging, which revealed that ~80% responded positively to drug stimulation while ~20% responded negatively. Immunofluorescence suggested that almost all cells were positive for the biomarkers. Immunofluorescence or qPCR indicates the biological characteristics on a molecular level, and they are usually performed in a relatively fixed condition with nonliving cells. There are methodological limitations to using fluorescence for quantification, so its use is mainly restricted to detecting the presence/absence of a biomarker and its subcellular location. Flow cytometry provides more precise information for quantification analysis compared to immunofluorescence. Calcium imaging reveals functional properties of cells as living entities, and cells are more likely to be influenced by transient environmental factors in calcium imaging, such as temperature, culture medium, and mechanical force posed by drug perfusion [48]. Therefore, poorly adapted cells, once out of the incubator, are more likely to show a negative response to glutamate or K^+^ stimulation. This may result in the homogeneity in immunofluorescence but heterogeneity in calcium imaging. Therefore, comprehensive analysis should be based on the results from multiple methods when considering induction efficiency.

Previous studies using human ESCs or iPS cells have reported much longer induction periods and generated fewer photoreceptors based on the positive expression rates of cell markers in immunofluorescence or flow cytometry [17–19, 24] (Table 1), and one study reports high generation rates of cones from human iPS cells, where 60-80% cone photoreceptors were generated by day 28 [49]. ~57.8% of the induced SHEDs expressed rhodopsin in flow cytometry, and ~60% more induced SHEDs responded positively to glutamate or high K^+^ than noninduced SHEDs on day 0. Considering the results of immunofluorescence, flow cytometry, and calcium imaging together, our study yielded ~60% photoreceptor-like cells, a relatively high output, and short induction period compared to ESCs and iPSCs (Table 1), and we considered this to demonstrate successful induction.

*In vivo* bioluminescent luciferase imaging showed 3 weeks of good survival after xenogenous transplantation, but further histological analysis suggested at least 3 months of sustainability *in vivo*. Similarly, there are reports of MSC sustainability on the basis of histological analysis for 2 weeks [9], or 6 weeks [10], or even 6 months [50] after transplantation in rodents with retinal degeneration. The difference of cell sustainability in histological analysis and *in vivo* bioluminescence imaging may be due to the methodology. First, *in vivo* imaging can be affected by many factors, such as slow blood circulation and metabolism in mice under general anesthesia and high background noise generated by the grey fur of animals, so sometimes a signal cannot be detected by this method. Second, dead cells are not easily eliminated from the subretinal space and may be identified in a tissue section, but they cannot be detected by *in vivo* imaging.

Transplanted SHEDs decreased considerably with time *in vivo.* It has been reported that retinal function improves and photoreceptors are rescued for 3 months [8, 9] or 5 months [10] even after transplanted MSCs are no longer detectable. The reasons for the therapeutic effects apparently lasting longer than transplanted cell survival *in vivo* is unclear so far and may be related to the multifunctional effects of MSCs such as neurotrophic, immunomodulatory, antiapoptotic, and angiogenic effects [11]. 3 weeks of robust cell viability by bioluminescence imaging and 3 months of cell sustainability based on histological evidence indicate preliminary success in using SHEDs to treat retinal degeneration.

## 5. Conclusions

We have demonstrated that SHEDs can differentiate into rod- and cone-like cells; the induced SHEDs display the characteristics of photoreceptors in morphology, as well as at the molecular and functional levels. In addition, luciferase-labeled SHEDs transplanted into the subretinal space of mice with retinal degeneration maintained good survival. The preliminary success in generating transplantable SHEDs and maintaining their survival *in vivo* raises the possibility of applying them to the treatment of retinal degeneration and opens a new avenue for further research on the therapeutic effects of SHEDs in mouse models of human disease.

## Figures and Tables

**Table 1:** Photoreceptor cells differentiation in previous studies.

Cells	Induction period	Photoreceptor precursor generation rate	Photoreceptor generation rates	Reference
Human ESCs	Over 3 weeks	~12%	<0.01% for rods and cones	Lamba et al., 2006 [17]
Human ESCs	130-170 days	~11.3-19.6%	~5.1-8.5% for rods; ~8.9-9.4% for cones	Osakada et al., 2008 [19]
Human ESCs/iPSCs	>80 days	~63.0%/14.4%	~46.4% for cones, unavailable for rods/44.6% for cones, unavailable for rods	Meyer et al., 2009 [18]
Human iPSCs	45 days	~16%	~18% for rods; 52-60% for cones	Mellough et al., 2012 [24]
Human ESCs	4-5 weeks	~70%	60%–80% for cones	Zhou et al., 2015 [49]

## Data Availability

The data associated with in vitro induction and in vivo transplantation used to support the findings of this study, including the results and the methods & materials, are included within the article or the supplementary materials. Detailed information about animal models is available upon request by contacting Dr. Liping Yang at alexlipingyang@bjmu.edu.cn.

## Supplementary Materials

Supplementary Materials Table S1: for antibodies used in immunostaining and flow cytometry. Table S2: for primers used in qPCR. Figure S1: immunostaining of retinal frozen sections from wild-type mice verifying the specificity of antibodies. a. Rhodopsin was positive in the outer segment (OS). b. Opsin was positive in the OS. c. Recoverin was positive in the cytoplasm of the outer nuclear layer (ONL) cells. d. PKC-*α* staining accumulated in the synaptic endings of inner nuclear layer (INL) cells (mainly bipolar cells). e, f. Representative images of negative controls without the primary antibodies (e) and using noninduced SHEDs for staining of retinal differentiation biomarkers (f). Scale bar: 20 *μ*m. Figure S2: a-b. Some induced SHEDs expressed the bipolar marker PKC-*α* (red arrow) on days 14-17, while others did not (white arrow). c-d. Induced SHEDs were positive for GFAP (c) on day 14 but negative (d) on day 24. Scale bar: 20 *μ*m. Figure S3: expression of the pluripotent biomarkers during the induction process. a. Noninduced SHEDs expressed the pluripotent marker SSEA4 on day 0. b. Flow cytometry plot showing that ~57.9% SHEDs expressed SSEA4. c. Representative image showing that induced SHEDs did not express SSEA4 on day 14. d. RT-qPCR analysis showing that NANOG and MYC were downregulated, and POU5F1 and SOX2 were upregulated after retinal induction. Scale bars: 20 *μ*m. Figure S4: histological images showing that no tumors formed in the retina after the transplantation of induced SHEDs. Scale bar: 40 *μ*m.

## References

[1] Jayakody S. A., Gonzalez-Cordero A., Ali R. R., Pearson R. A. (2015). Cellular strategies for retinal repair by photoreceptor replacement. *Progress in Retinal and Eye Research*.

[2] MacLaren R. E., Pearson R. A. (2007). Stem cell therapy and the retina. *Eye*.

[3] Caplan A. I., Correa D. (2011). The MSC: an injury drugstore. *Cell Stem Cell*.

[4] Keating A. (2012). Mesenchymal stromal cells: new directions. *Cell Stem Cell*.

[5] Miura M., Gronthos S., Zhao M. (2003). SHED: stem cells from human exfoliated deciduous teeth. *Proceedings of the National Academy of Sciences of the United States of America*.

[6] Kerkis I., Kerkis A., Dozortsev D. (2006). Isolation and characterization of a population of immature dental pulp stem cells expressing OCT-4 and other embryonic stem cell markers. *Cells Tissues Organs*.

[7] Yamaza T., Kentaro A., Chen C. (2010). Immunomodulatory properties of stem cells from human exfoliated deciduous teeth. *Stem Cell Research & Therapy*.

[8] Lu B., Wang S., Girman S., McGill T., Ragaglia V., Lund R. (2010). Human adult bone marrow-derived somatic cells rescue vision in a rodent model of retinal degeneration. *Experimental Eye Research*.

[9] Tzameret A., Sher I., Belkin M. (2014). Transplantation of human bone marrow mesenchymal stem cells as a thin subretinal layer ameliorates retinal degeneration in a rat model of retinal dystrophy. *Experimental Eye Research*.

[10] Tzameret A., Sher I., Belkin M. (2015). Epiretinal transplantation of human bone marrow mesenchymal stem cells rescues retinal and vision function in a rat model of retinal degeneration. *Stem Cell Research*.

[11] Ding S. L. S., Kumar S., Mok P. L. (2017). Cellular reparative mechanisms of mesenchymal stem cells for retinal diseases. *International Journal of Molecular Sciences*.

[12] Taghipour Z., Karbalaie K., Kiani A. (2012). Transplantation of undifferentiated and induced human exfoliated deciduous teeth-derived stem cells promote functional recovery of rat spinal cord contusion injury model. *Stem Cells and Development*.

[13] Inoue T., Sugiyama M., Hattori H., Wakita H., Wakabayashi T., Ueda M. (2013). Stem cells from human exfoliated deciduous tooth-derived conditioned medium enhance recovery of focal cerebral ischemia in rats. *Tissue Engineering Part A*.

[14] Sugimura-Wakayama Y., Katagiri W., Osugi M. (2015). Peripheral nerve regeneration by secretomes of stem cells from human exfoliated deciduous teeth. *Stem Cells and Development*.

[15] Shimojima C., Takeuchi H., Jin S. (2016). Conditioned medium from the stem cells of human exfoliated deciduous teeth ameliorates experimental autoimmune encephalomyelitis. *The Journal of Immunology*.

[16] Sugiyama M., Iohara K., Wakita H. (2011). Dental pulp-derived CD31^−^/CD146^−^ side population stem/progenitor cells enhance recovery of focal cerebral ischemia in rats. *Tissue Engineering Part A*.

[17] Lamba D. A., Karl M. O., Ware C. B., Reh T. A. (2006). Efficient generation of retinal progenitor cells from human embryonic stem cells. *Proceedings of the National Academy of Sciences of the United States of America*.

[18] Meyer J. S., Shearer R. L., Capowski E. E. (2009). Modeling early retinal development with human embryonic and induced pluripotent stem cells. *Proceedings of the National Academy of Sciences of the United States of America*.

[19] Osakada F., Ikeda H., Mandai M. (2008). Toward the generation of rod and cone photoreceptors from mouse, monkey and human embryonic stem cells. *Nature Biotechnology*.

[20] Hong D.-H., Pawlyk B. S., Shang J., Sandberg M. A., Berson E. L., Li T. (2000). A retinitis pigmentosa GTPase regulator (RPGR)- deficient mouse model for X-linked retinitis pigmentosa (RP3). *Proceedings of the National Academy of Sciences of the United States of America*.

[21] Huang W. C., Wright A. F., Roman A. J. (2012). RPGR-associated retinal degeneration in human X-linked RP and a murine model. *Investigative Ophthalmology & Visual Science*.

[22] Bachiller D., Klingensmith J., Kemp C. (2000). The organizer factors chordin and noggin are required for mouse forebrain development. *Nature*.

[23] Mukhopadhyay M., Shtrom S., Rodriguez-Esteban C. (2001). *Dickkopf1* is required for embryonic head induction and limb morphogenesis in the mouse. *Developmental Cell*.

[24] Mellough C. B., Sernagor E., Moreno-Gimeno I., Steel D. H. W., Lako M. (2012). Efficient stage-specific differentiation of human pluripotent stem cells toward retinal photoreceptor cells. *Stem Cells*.

[25] Watanabe K., Kamiya D., Nishiyama A. (2005). Directed differentiation of telencephalic precursors from embryonic stem cells. *Nature Neuroscience*.

[26] Kelley M. W., Turner J. K., Reh T. A. (1995). Regulation of proliferation and photoreceptor differentiation in fetal human retinal cell cultures. *Investigative Ophthalmology & Visual Science*.

[27] Kopp J. L., Ormsbee B. D., Desler M., Rizzino A. (2008). Small increases in the level of Sox2 trigger the differentiation of mouse embryonic stem cells. *Stem Cells*.

[28] Gao Z., Cox J. L., Gilmore J. M. (2012). Determination of protein interactome of transcription factor Sox2 in embryonic stem cells engineered for inducible expression of four reprogramming factors. *Journal of Biological Chemistry*.

[29] Cartwright P., McLean C., Sheppard A., Rivett D., Jones K., Dalton S. (2005). LIF/STAT3 controls ES cell self-renewal and pluripotency by a Myc-dependent mechanism. *Development*.

[30] Chambers I., Silva J., Colby D. (2007). Nanog safeguards pluripotency and mediates germline development. *Nature*.

[31] Lankford K. L., Letourneau P. C. (1989). Evidence that calcium may control neurite outgrowth by regulating the stability of actin filaments. *Journal of Cell Biology*.

[32] Catterall W. A., Few A. P. (2008). Calcium channel regulation and presynaptic plasticity. *Neuron*.

[33] Neher E., Sakaba T. (2008). Multiple roles of calcium ions in the regulation of neurotransmitter release. *Neuron*.

[34] Liu S. J., Zukin R. S. (2007). Ca^2+^-permeable AMPA receptors in synaptic plasticity and neuronal death. *Trends in Neurosciences*.

[35] Coffelt S. B., Marini F. C., Watson K. (2009). The pro-inflammatory peptide LL-37 promotes ovarian tumor progression through recruitment of multipotent mesenchymal stromal cells. *Proceedings of the National Academy of Sciences of the United States of America*.

[36] Toyoshima M., Tanaka Y., Matumoto M. (2009). Generation of a syngeneic mouse model to study the intraperitoneal dissemination of ovarian cancer with *in vivo* luciferase imaging. *Luminescence*.

[37] Wang H., Cao F., de A. (2009). Trafficking mesenchymal stem cell engraftment and differentiation in tumor-bearing mice by bioluminescence imaging. *Stem Cells*.

[38] Hung S.‐. C., Cheng H., Pan C.‐. Y., Tsai M. J., Kao L.‐. S., Ma H.‐. L. (2002). In vitro differentiation of size-sieved stem cells into electrically active neural cells. *Stem Cells*.

[39] Kohyama J., Abe H., Shimazaki T. (2001). Brain from bone: efficient "meta-differentiation" of marrow stroma-derived mature osteoblasts to neurons with noggin or a demethylating agent. *Differentiation*.

[40] Fox L. E., Shen J., Ma K. (2010). Membrane properties of neuron-like cells generated from adult human bone-marrow-derived mesenchymal stem cells. *Stem Cells and Development*.

[41] Tucker B. A., Park I. H., Qi S. D. (2011). Transplantation of adult mouse iPS cell-derived photoreceptor precursors restores retinal structure and function in degenerative mice. *PLoS One*.

[42] Homma K., Okamoto S., Mandai M. (2013). Developing rods transplanted into the degenerating retina of Crx-knockout mice exhibit neural activity similar to native photoreceptors. *Stem Cells*.

[43] Grünert U., Haverkamp S., Fletcher E. L., Wässle H. (2002). Synaptic distribution of ionotropic glutamate receptors in the inner plexiform layer of the primate retina. *The Journal of Comparative Neurology*.

[44] Hack I., Frech M., Dick O., Peichl L., Brandstätter J. H. (2001). Heterogeneous distribution of AMPA glutamate receptor subunits at the photoreceptor synapses of rodent retina. *European Journal of Neuroscience*.

[45] Hanna M. C., Calkins D. J. (2007). Expression of genes encoding glutamate receptors and transporters in rod and cone bipolar cells of the primate retina determined by single-cell polymerase chain reaction. *Molecular Vision*.

[46] Chu D. T., Malinowska E., Gawronska-Kozak B., Kozak L. P. (2014). Expression of adipocyte biomarkers in a primary cell culture models reflects preweaning adipobiology. *The Journal of Biological Chemistry*.

[47] Colter D. C., Sekiya I., Prockop D. J. (2001). Identification of a subpopulation of rapidly self-renewing and multipotential adult stem cells in colonies of human marrow stromal cells. *Proceedings of the National Academy of Sciences of the United States of America*.

[48] Bootman M. D., Rietdorf K., Collins T., Walker S., Sanderson M. (2013). Loading fluorescent Ca^2+^ indicators into living cells. *Cold Spring Harbor Protocols*.

[49] Zhou S., Flamier A., Abdouh M. (2015). Differentiation of human embryonic stem cells into cone photoreceptors through simultaneous inhibition of BMP, TGF*β* and Wnt signaling. *Development*.

[50] Xian B., Zhang Y., Peng Y. (2016). Adult human peripheral blood mononuclear cells are capable of producing neurocyte or photoreceptor-like cells that survive in mouse eyes after preinduction with neonatal retina. *Stem Cells Translational Medicine*.

