# Supplementary material for: Differentiation of Stem Cells from Human Exfoliated Deciduous Teeth into Retinal Photoreceptor-Like Cells and Their Sustainability In Vivo
Source: Stem Cells Int. 2019 Feb 14;2019:2562981. doi: 10.1155/2019/2562981 (PMC6393909; doi:10.1155/2019/2562981)
Supplement: Supplementary Materials — Table S1: for antibodies used in immunostaining and flow cytometry. Table S2: for primers used in qPCR. Figure S1: immunostaining of retinal frozen sections from wild-type mice verifying the specificity of antibodies. a. Rhodopsin was positive in the outer segment (OS). b. Opsin was positive in the OS. c. Recoverin was positive in the cytoplasm of the outer nuclear layer (ONL) cells. d. PKC-α staining accumulated in the synaptic endings of inner nuclear layer (INL) cells (mainly bipolar cells). e, f. Representative images of negative controls without the primary antibodies (e) and using noninduced SHEDs for staining of retinal differentiation biomarkers (f). Scale bar: 20 μm. Figure S2: a-b. Some induced SHEDs expressed the bipolar marker PKC-α (red arrow) on days 14-17, while others did not (white arrow). c-d. Induced SHEDs were positive for GFAP (c) on day 14 but negative (d) on day 24. Scale bar: 20 μm. Figure S3: expression of the pluripotent biomarkers during the induction process. a. Noninduced SHEDs expressed the pluripotent marker SSEA4 on day 0. b. Flow cytometry plot showing that ~57.9% SHEDs expressed SSEA4. c. Representative image showing that induced SHEDs did not express SSEA4 on day 14. d. RT-qPCR analysis showing that NANOG and MYC were downregulated, and POU5F1 and SOX2 were upregulated after retinal induction. Scale bars: 20 μm. Figure S4: histological images showing that no tumors formed in the retina after the transplantation of induced SHEDs. Scale bar: 40 μm. [file 2562981.f1.doc]

**Supplementary Material**

Table S1: Antibodies used for immunofluorescence (IF) and flow cytometry (FC)

| Antigen | Host | Catalog Number | Supplier | Dilution | Application |
| --- | --- | --- | --- | --- | --- |
| Nestin | mouse | ab22035 | Abcam | 1:100 | IF |
| Nestin | mouse | MAB1259 | R&D | 1:100 | FC |
| β-III tubulin | rabbit | ab18207 | Abcam | 1:800/1:100 | IF/FC |
| SSEA4 | mouse | Ab16287 | Abcam | 1:40/1:100 | IF//FC |
| GFAP | mouse | 3670T | CST | 1:300 | IF |
| GFAP | mouse | 561483 | BD | 1:20 | FC |
| Tau | mouse | HT7 | Invitrogen | 1:500 | IF |
| PKC-α | rabbit | 2056T | CST | 1:100/1:50 | IF/FC |
| Recoverin | rabbit | 10073-1-AP | Proteintech | 1:400/1:200 | IF/FC |
| Rhodopsin | mouse | MA1-722 | Invitrogen | 1:100/1:100 | IF/FC |
| L/M- Opsin | rabbit | AB5405 | Merck Millipore | 1:200 | IF |
| AIPL1 | rabbit | 15108-1-AP | Proteintech | 1:400 | IF |
| OTX2 | rabbit | 13497-1-AP | Proteintech | 1:50 | IF |
| GluR2 | mouse | 810501 | Biolegend | 1:400 | IF |
| CD34 | mouse | 560941 | BD | 1:5 | FC |
| CD45 | mouse | 560975 | BD | 1:5 | FC |
| CD90 | mouse | 561969 | BD | 1：100 | FC |
| CD73 | mouse | 561014 | BD | 1:5 | FC |
| CD105 | mouse | 561443 | BD | 1:20 | FC |
| CD146 | mouse | 561013 | BD | 1:5 | FC |

Table S2: Primers used for qPCR

| Gene name | Forward (5`-3`) | Reverse (5`-3`) | Amplicon size (bp) | Genbank accession |
| --- | --- | --- | --- | --- |
| NANOG | TGGAAACGTCTGCTAAGACTGC | GCTGAGGGCCAATTTTAATGCC | 97 | NM_024865 |
| SOX2 | GCCGAGTGGAAACTTTTGTCG | GGCAGCGTGTACTTATCCTTCT | 155 | NM_003106 |
| POU5F1 | AGCTTGGATCTCAGGGTCAC | TGGCTGAATACCTTCCCTGG | 187 | Z11898 |
| MYC | GGCTCCTGGCAAAAGGTCA | CTGCGTAGTTGTGCTGATGT | 119 | NM_002467 |
| NES | CTGCTACCCTTGAGACACCTG | GGGCTCTGATCTCTGCATCTAC | 141 | NM_006617 |
| PAX6 | CCTCATTTCCCGCTCTGGTT | TCTCAGATTCCTATGCTGATTGGT | 111 | NM_001604 |
| RAX | AAGCCCCTCGACCCTACTG | CCGCCGATGCTTTTTCTTGG | 128 | NM_013435 |
| VSX2 | GGCGAACACGGACAATCTTTA | GGCAGCTCCGTTTTCATGG | 118 | NM_182894 |
| RCVRN | CCAGAGCATCTACGCCAAGTT | CCGTCGAGGTTGGAATCGAAG | 90 | NM_002903 |
| AIPL1 | GTTGATGCCCCGAGTGATTAC | TGAAGAGCCGATTTCCCTCTC | 103 | NM_001033055 |
| ASCL1 | CCCAAGCAAGTCAAGCGACA | AAGCCGCTGAAGTTGAGCC | 81 | NM_004316 |
| NRL | GGCTCCACACCTTACAGCTC | GGCCCATCAACAGGGACTG | 212 | NM_006177 |
| CRX | CGGCCTAGACCCCTACCTTT | GCCATAGCTCTGGCCTGATA | 130 | NM_000554 |
| NEUROD1 | GTCTCCTTCGTTCAGACGCTT | AAAGTCCGAGGATTGAGTTGC | 89 | NM_002500 |
| OTX2 | CAAAGTGAGACCTGCCAAAAAGA | TGGACAAGGGATCTGACAGTG | 179 | NM_172337 |
| RHO | GTGCCCTTCTCCAATGCGA | TGAGGAAGTTGATGGGGAAGC | 142 | NM_006583 |
| OPN1SW | ATGGGCCTCAGTACCACATTG | GGGAACCCTATAAGGAAGACAGT | 85 | NM_001708 |
| GAPDH | GGAGCGAGATCCCTCC AAAAT | GGCTGTTGTCATACTTCTCATGG | 197 | NM_001256799 |


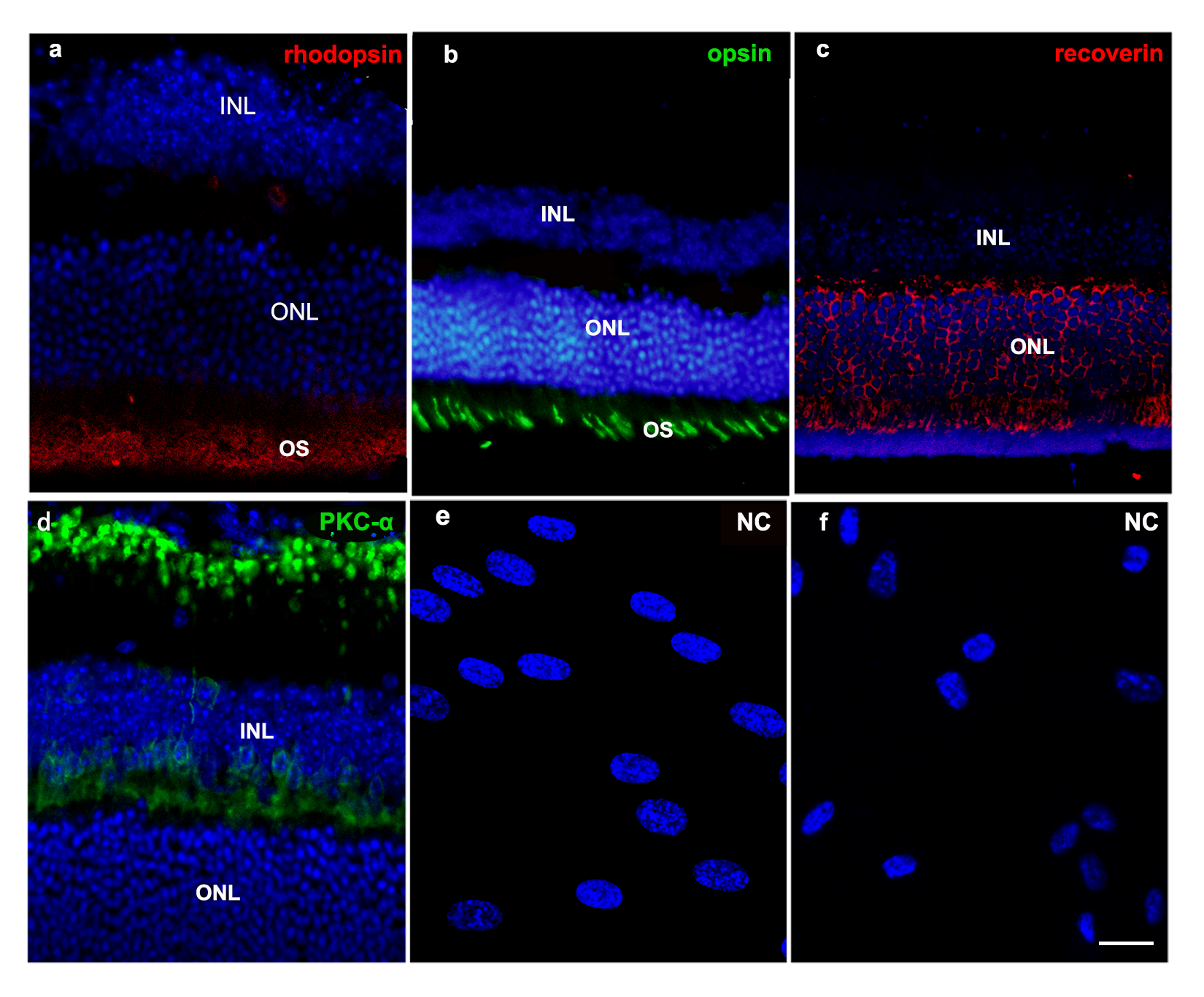


Figure S1. Immuno-staining of retinal frozen sections from wild-type mice verifying the specificity of antibodies. a. Rhodopsin was positive in the outer segment (OS). b. Opsin was positive in the OS. c. Recoverin was positive in the cytoplasm of the outer nuclear layer (ONL) cells. d. PKC-α staining accumulated in the synaptic endings of inner nuclear layer (INL) cells (mainly bipolar cells). e, f. Representative images of negative controls without the primary antibodies (e) and using non-induce SHEDs for staining of retinal differentiation biomarkers (f). *Scale bar: 20 μm.*


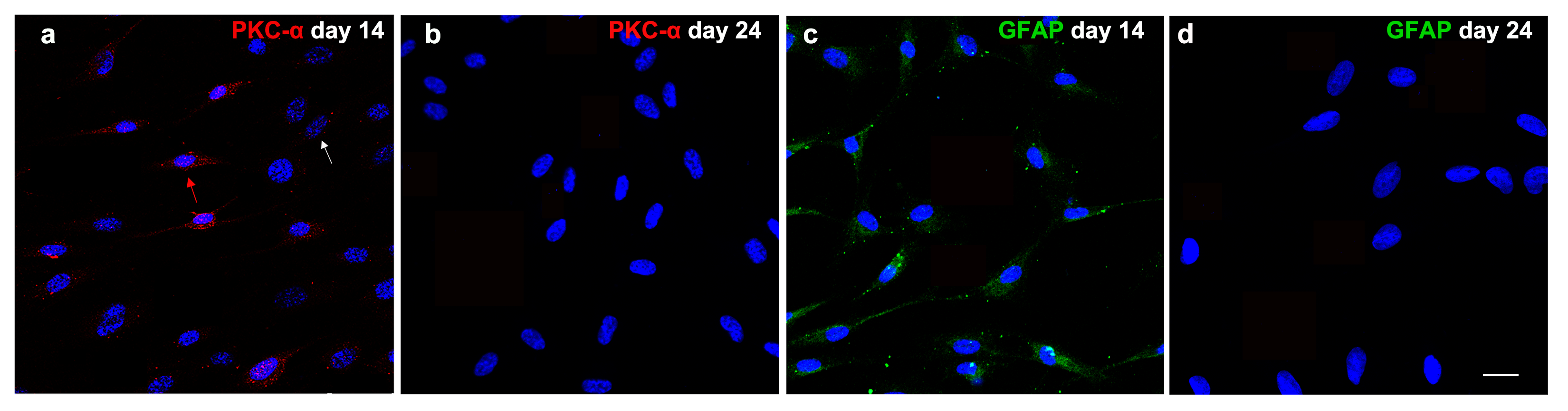


Figure S2. a-b. Some induced SHEDs expressed the bipolar marker PKC-α (red arrow) on days 14-17, while others did not (white arrow). c-d. Induced SHEDs were positive for GFAP (c) on day 14 but negative (d) on day 24. *Scale bar: 20 μm.*

*
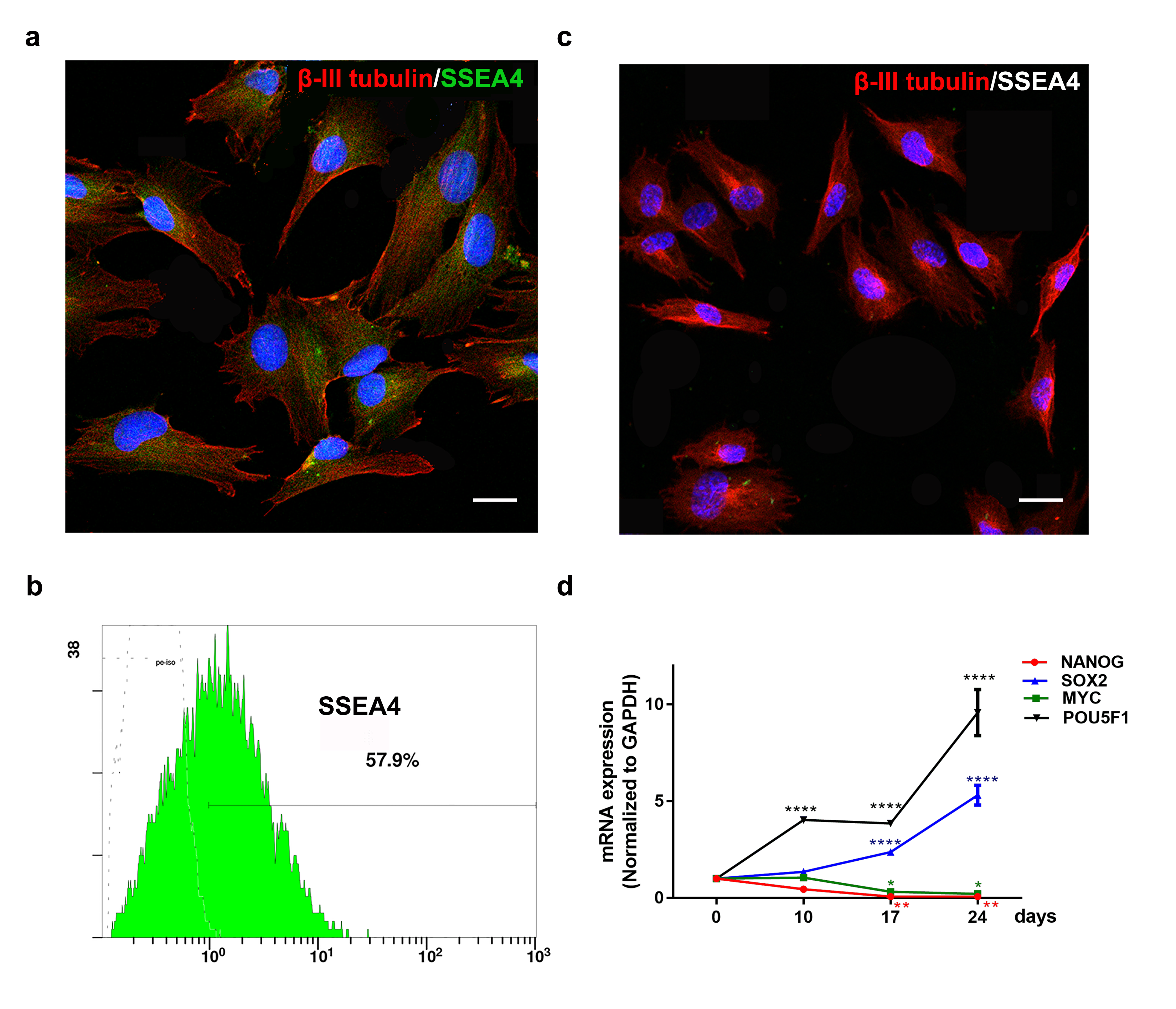
*

Figure S3. Expression of the pluripotent biomarkers during the induction process. a. Non-induced SHEDs expressed the pluripotent marker SSEA4 on day 0. b. Flow cytometry plot showing that ~57.9% SHEDs expressed SSEA4. c. Representative image showing that induced SHEDs did not express SSEA4 on day 14. d. RT-qPCR analysis showing that NANOG and MYC were downregulated, and POU5F1 and SOX2 were upregulated after retinal induction. *Scale bars: 20 μm.*

*
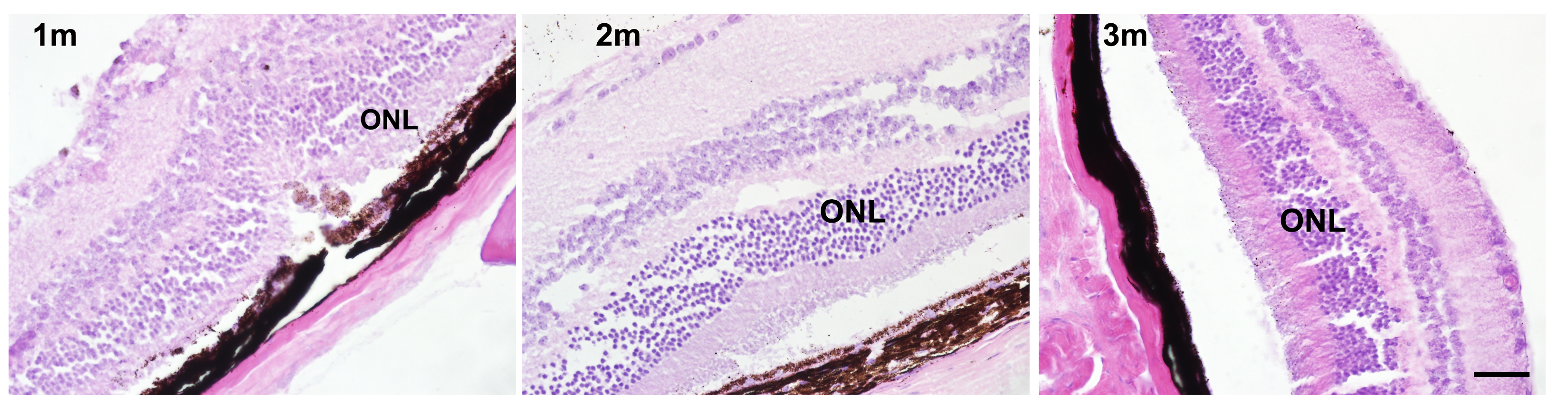
*

Figure S4. Histological images showing that no tumors formed in the retina after the transplantation of induced SHEDs. *Scale bar: 40 μm.*
